# Paralytic rabies outbreak mimicking guillain–Barré syndrome in French Amazonia

**DOI:** 10.1371/journal.pntd.0014149

**Published:** 2026-03-30

**Authors:** Nathalie Deschamps, Claire Mayence, Perrine Parize, Maylis Douine, Clémentine Montagnac, Stephanie Houcke, Loïc Epelboin, Amina Nasri, Felix Djossou, Florence Larrous, Katherine Worsley-Tonks, Karim Hamiche, Alessandra Monaya, Cyril Rousseau, Samuel Gavohedo, Kinan Drak Alsibai, Magalie Demar, Bertrand de Toffol, Hervé Bourhy, Hatem Kallel

**Affiliations:** 1 Neurology Department, University Hospital of Guyane, Cayenne, French Guiana, France; 2 Département de Recherche et d’Innovation en Santé Publique (DRISP), Centre d’Investigation Clinique Inserm 1424) University Hospital of Guyane, Cayenne, French Guiana, France; 3 Intensive Care Unit, University Hospital of Guyane, Cayenne, French Guiana, France; 4 Institut Pasteur, Université Paris Cité, Unit Lyssavirus Epidemiology and Neuropathology, National Reference centre for rabies, European Reference Laboratory for Emerging, rodent born and zoonotic viral diseases (EURL-ERZV), WHO rabies collaborating centre, Institut Pasteur, Paris, France; 5 Infectious and Tropical Diseases Unit, University Hospital of Guyane, Cayenne, French Guiana, France; 6 Tropical Biome and immunopathology CNRS UMR-9017, Inserm U, Université de Guyane, French Guiana, France; 7 Forensic Medical Unit, University Hospital of Guyane, Cayenne, French Guiana, France; 8 Radiology Department, University Hospital of Guyane, Cayenne, French Guiana, France; 9 Remote Health Care Centers, University Hospital of Guyane, Cayenne, French Guiana, France; 10 Department of Pathology, University Hospital of Guyane, Cayenne, French Guiana, France; 11 Laboratory Department, University Hospital of Guyane, Cayenne, French Guiana, France; Shanghai Jiao Tong University, CHINA

## Abstract

**Background:**

In the Amazonian region, vampire bats are the primary reservoir of rabies virus, causing sporadic and lethal human rabies cases that often remain unnoticed. Managing human cases in this region is challenging and further complicated by atypical clinical forms and the potential exposure to various toxic compounds, particularly among gold miners.

**Methods:**

We carried out clinical, electrical, biological and histological analysis of concurrent cases of progressive motor neuronopathy and fatal encephalitis in a context of regular exposure to bat bites of gold miners living in a small and remote gold mine camp in Amazonia, in French Guiana, South America.

**Findings:**

We analyzed a spatio-temporal cluster of three suspected rabies cases in 2024 with a fatal outcome, with concomitant onset of acute bilateral lower-limb paralysis without demyelination, two of which occurred presumably two weeks after a bat-bite. Electroneuromyography suggested the involvement of the anterior horn of the spinal cord, as described in furious forms of rabies. None of the cases exhibited other cardinal signs of the furious form. Confirmation of rabies was obtained for them on sera and brain biopsies collected ante- and post-mortem respectively.

**Interpretation:**

The concurrent occurrence of disease, the axonal motor neuropathy mimicking the motor form of Guillain Barré syndrome in the context of paralytic rabies, lead to diagnostic-wandering. This underscores the importance of thinking about vampire bat rabies virus in the presence of any atypical neurological picture in patients living in exposed areas in Latin America.

## Author summary

In the Amazon region, certain bats known as vampire bats are the main reservoir for the rabies virus. They can transmit the disease to humans through bites. Although rare, human rabies is almost always fatal and can go unnoticed, particularly in isolated areas. Diagnosis is made all the more difficult by the fact that the disease can take unusual forms and that certain populations, such as gold miners, are also exposed to toxic substances that can cause neurological disorders.

This study reports three cases that occurred in 2024 among gold miners living in an isolated mining camp in French Guiana. These patients developed almost simultaneously rapid paralysis of both legs, followed by severe brain damage leading to death. Two of them had been bitten by bats about two weeks earlier. Neurological examinations showed damage to the spinal cord resembling certain forms of rabies, but without the usual signs of the disease, which delayed diagnosis. Rabies was finally confirmed by biological tests before and after death.

These cases show that in the Amazon region, any unusual neurological damage should raise suspicion of rabies transmitted by bats, in order to improve diagnosis and treatment.

## Introduction

Rabies is a zoonotic disease caused by a neurotropic Lyssavirus leading to acute almost invariably fatal encephalitis. It causes about 59,000 human deaths annually, mainly in Asia and Africa [1]. Among mammals, carnivores and bats are the primary reservoirs with 99% of human cases globally linked to dog bites [2]. However, in the Americas, bats have become the main source of infection following substantial success in control of dog bite-associated rabies [3].

In Latin America, the common vampire bat (*Desmodus rotundus)* is the principal wildlife reservoir [4]. Its bites transmit the virus to livestock and occasionally to human leading to significant economic losses in cattle production and public health burdens [5–9]. The virus, present in the saliva is mainly transmitted through feeding bites [10]. Human attacks are uncommon but may increase with deforestation, agricultural expansion, and human encroachment into bat habitats [11].

Recent years have seen outbreaks of bat-transmitted rabies in the Amazon, particularly in Peru, Venezuela, and Brazil [5,12]. As dog rabies declines, vampire bat rabies virus is emerging as a major zoonotic concern in this region [8,13]. Human cases occur mainly in isolated rural or communities with limited healthcare access and poor awareness of preventive measures [14]. In South America, those most at risk include illegal gold miners “garimpos”, loggers, and gold prospectors living in precarious conditions, that facilitate bat exposure [3]. French Guiana, a French territory bordered by Brazil and Suriname, is heavily affected by illegal gold mining within its vast Amazon rainforest, home to an estimated 10,000 illegal gold miners from northern Brazil [15].

Clinically, rabies presents as either a “furious” encephalitic or a “paralytic” form [16,17]. The latter form is often linked to *Desmodus* strains, may mimic Guillain-Barré syndrome (GBS), complicating diagnosis [18,19]. Here, we analyzed a cluster of three laboratory-confirmed cases of rabies among illegal gold miners in a remote informal mining site in French Guiana all presenting atypical lower-limb motor deficits without any associated demyelination, highlighting the urgent need to improve rabies recognition and prevention in vulnerable Amazonian populations.

## Methods

### Ethics statement

We obtained written consent from the families of the three confirmed cases. The European General Data Protection Regulation (GRPD) does not apply to the processing of personal data of deceased people. Article 86 of the French Data Protection Act states that information concerning deceased persons, including that contained in certificates of cause of death, “may be processed for the purposes of research, study or evaluation in the field of health, unless the person concerned has, during his or her lifetime, expressed his or her refusal in writing.” The study was, therefore, an internal research study, in accordance with the Commission Nationale de l’Informatique et des Libertés (CNIL) definition and complied with the European General Data Protection Regulation (GDPR). According to French and European regulations (Regulation 2016/679 General Data Protection Regulation) and the typology of the study (Articles 44. 1 and 65.2 of the French Data Protection Act and Article and L1110-12 of the French Public Health Code), individual information and consent of each participant is not required.

This is a descriptive observational study of three confirmed rabies cases hospitalized at the Cayenne hospital in French Guiana in February 2024. We collected and analyzed data including neurological examination, motor and sensitive conduction analysis by electroneuromyogram (ENMG), infectiological, toxicological and immunological work-up tests, histological and imaging analysis to rule out differential diagnoses.

### Laboratory diagnosis of rabies

Biological samples taken from the three cases were analyzed at the French National Reference center for rabies, European Reference Laboratory for Emerging, rodent-borne and zoonotic viral diseases (EURL-ERBZV) and World Health Organization (WHO) rabies collaborating center, Institut Pasteur, Paris, France (Fig 2).

*Ante-mortem* diagnosis of human rabies was based on viral RNA detection by RT-PCR from a skin biopsy collected in a richly innervated area (preferably at the base of the neck in an area rich in hair follicles) and on three saliva samples, collected sequentially on at least three occasions, with a 3–6 hour interval between samples, by swabbing or direct collection [20,21]. The presence of viral-specific antibodies was also tested in CSF and serum of Cases 1 and 2 by a modified rapid fluorescent focus inhibition test (RFFIT), recommended as the reference technique by WHO [22]. Rabies virus-neutralizing antibody titers were calculated by comparing with a reference serum calibrated to the WHO reference serum [23]. The threshold of detection was 0·1 IU/mL [24].

After the three patients’ death, brain biopsies were collected from the occipital, parietal, frontal and temporal cerebellum, hippocampus and brainstem, and were analyzed by histochemistry. *Post-mortem* rabies diagnosis was based on the presence of viral antigens in brain biopsies (fluorescent antibody test), the isolation of virus in cell culture and/or the presence of viral nucleic acids in samples (RT-PCR) depending on the state of the samples submitted (frozen at -80°C or fixed in 4% paraformaldehyde), *post-mortem* diagnosis of rabies was based on detecting rabies virus antigens by direct fluorescent antibody staining of brain samples using anti-rabies Nucleocapsid conjugate (Bio-Rad ref 3572112), isolation of virus in neuroblastoma cells (N2A), and/or the presence of viral nucleic acid by RT-PCR using primers and techniques previously described [25].

### Characterization of rabies virus strains

To obtain whole-genome sequences of the rabies virus, total RNA was extracted with a Direct-zol RNA miniprep kit (Zymo Research) from the brain biopsies (approximately 0.5 cm3 each) and purified using Agencourt RNA Clean XP beads (Beckman Coulter at 1:1.8 ratio). The RNA samples were then processed for next generation sequencing (NGS) as described elsewhere [20,26]. NGS data were analyzed using *de novo* assembly and mapping using CLC (Assembly Cell), with a dedicated workflow built on the Institut Pasteur Galaxy platform [27] The quality and the accuracy of the final genome sequences were checked after a final mapping step of the original cleaned reads and visualized using IGV (version 2.17.4) [28]. Identification of the open reading frames was performed using SnapGene software version 8.0.2.

In addition to the two newly generated sequences generated as part of this study, whole-genome sequences were downloaded from the NCBI Virus database [29]. A multiple sequence alignment was constructed using MAFFT version 7.490 with default parameters [30] and subsequently visually inspected for quality in MEGA version 11 [31]. A maximum likelihood phylogenetic tree was generated in IQ-TREE version 2.3.5 [32] and the best fit model was identified using ModelFinder [33]. Node support was assessed using 1,000 bootstrap replicates. The consensus tree was visualized and annotated using FigTree version 1.4.4 (http://tree.bio.ed.ac.uk/software/figtree/).

## Results

### Clinical presentation

**Fig 1:**
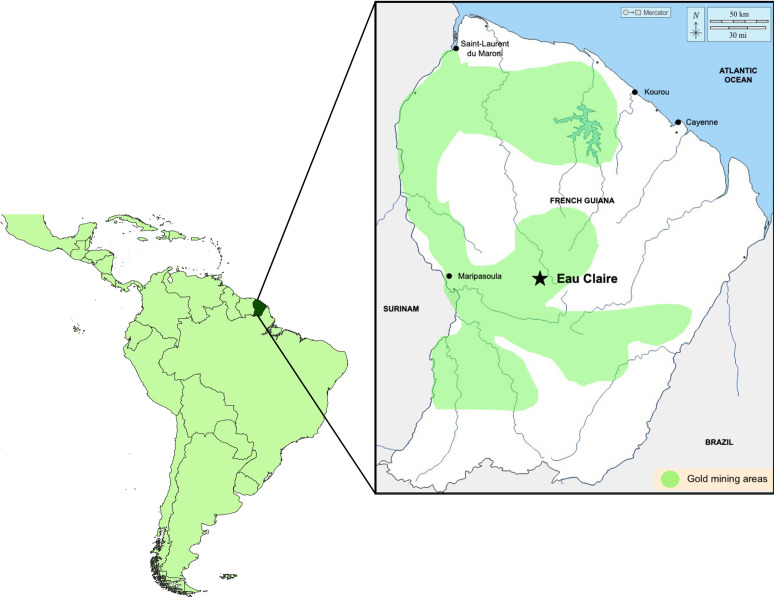
Map of French Guiana and gold mining area. Part of the map was created using QGIS software. The background dataset are Natural Earth Data (https://www.naturalearthdata.com/downloads/10m-cultural-vectors/); the terms of use: http://www.naturalearthdata.com/about/terms-of-use/. The zoomed-in part is not licensed and is a reproduction of an article by Maylis Douine and al.[48] (Map back-ground from dmap: https://www.d-maps.com/carte.php?num_car=15480&lang=fr; the terms of use:https://www.d-maps.com/conditions.php?lang=fr).

Three unrelated Brazilian patients from Macapa and Maranho in northern Brazil were working in clandestine gold mines. They were working on an illegal site called “Eau Claire”(Fig 1), located in the Maripasoula municipality, in the French Amazon region, with a group of around 25 people. This mining camp is extremely remote, located approximately two to four days away from the nearest health center by boat and on foot. The site is home to many vampire bats, snakes, dogs and cats. They lived in precarious conditions without drinking water, slept in hammocks and worked every day from 5am to 7pm. They regularly suffered from joint pain and leishmaniasis, for which they self-medicated with drugs from Brazil and provided by an on-site medicine shop.

Once the first symptoms were observed, Case 1 and 2 waited between two and three days before being transported by pirogue to the remote health care center (RHCC) in Maripasoula. The journey took around two days. Twenty-four hours after being admitted to the RHCC, they were transferred to the emergency unit in Cayenne by helicopter. Case 3 was transferred directly to Cayenne Hospital without going through the RHCC. None of these patients received a rabies post exposure prophylaxis nor had a previous history of rabies vaccination.

The three cases were described in Table 1. All paraclinical examination were taken at Cayenne Hospital.

**Table 1:** Description of three confirmed cases rabies.

	Case 1	Case 2	Case 3
Sex	Male	Male	Male
Age	51 years old	43 years old	23 years old
Nationality	Brazilian	Brazilian	Brazilian
Illegal Miner	Yes	Yes	Yes
Medical history	Back pain treated with benzylpenicillin and intramuscular betamethasone	Back pain treated with intramuscular betamethasone	Cutaneous leishmaniasis, treated with pentamidine injection and another unidentified molecule
Bite	Vampire bat bite 2 weeks before symptom	Vampire bat bite 2 weeks before symptom	No bite reported
Symptoms	Hypoesthesia of the lower limbs Progressed to bilateral motor weakness	Hypoesthesia of the lower limb Progressed to bilateral motor weakness Urinary retention	Motor weakness in left lower limb Progessing to bilateral involvement Acute urinary retention
Fever	No	No	No
Autonomic dysfunction	No	No	No
Delay to consultation	7 days	8 days	12 days
Neurological examination	Sensory and motor deficit in lower limbs (2/5 MRC) Tendon reflex present	Motor deficit (2/5 MRC) in lower limbs Tendon reflex present	Motor weakness (1/5 MRC) in lower limbs Areflexia
Delay to coma	8 days	9 days	13 days
Diagnosis hypothesis	Guillain-Barre syndrome Infectious encephalitis	Guillain-Barre syndrome Infectious encephalitis	Infectious encephalitis
Administered treatment	IgIV, cefotaxime, acyclovir, amoxicillin	IgIV, cefotaxime, acyclovir, amoxicillin	cefotaxime, acyclovir, amoxicillin
Delay to death	18 days	21 days	16 days
			
Biology			
Blood			
-Infectious diseases work-up	Negative	Negative	Negative
-Toxicology work-up	Negative	Negative	Negative
-antigangliosides antibodies	Serum: Negative CSF: Negative	Serum: Negative CSF: Negative	CSF: Negative
-Glucose (mmol/L)	NA	NA	7.5
CSF			
Cells (/mm3)	360	120	50
-Lymphocytes (%)	87	99	81
-Neutrophils (%)	0	1	15
-Monocytes (%)	13	0	4
Protein (g/L)	1·6	1·31	0·99
Glucose (mmol/L)	3·3	2·95	3·72
Lactates (mmol/L)	4·1	5·3	3·9
			
Electroneuromyogram	Motor: axonal involvement Sensory: normal	Motor: axonal involvement Sensory: normal	Motor: axonal involvement Sensory: normal
			
Electroencephalogram	Slow theta rhythm without epileptic abnormalities.	Slow theta rhythm without epileptic abnormalities.	Slow theta rhythm without epileptic abnormalities
			
Imaging	Brain scan: normal	Brain scan: normal	Brain MRI: spinal T2 hyperintensity, periaqueductal, bilateral thalami FLAIR hyperintensities, and diffusion hyperintensity with restriction of ADC of the bilateral frontal cortical ribbon and internal capsule not gadolinium enhanced

Legend: CSF: cerebrospinal fluid; MRC: Medical Concil Research; Not available

Infectious diseases workup included in blood PCR HIV, HTLV1 and 2, HBV, HCV, HHV6, VZV, dengue fever, Zika, Chikungunya, parvo B19, rickettsiosis, toxocariasis, toxoplasmosis, EBV, leptospirosis, malaria, pneumococcus, legionella disease; and in CSF: PCR cryptococcus, Listeria monocytogenes, Neisseria meningitidis, Streptococcus agalactiae, Streptococcus pneumoniae, Mycoplasma pneumoniae, Streptococcus pyogenes, herpes simplex virus, cytomegalovirus, serology of dengue virus, Zika virus, chikungunya virus, yellow fever virus, Zika, West-Nile virus, and mycology analysis; in feces: botulinum toxin, enterovirus). Toxicology workup included determination of copper, lead, mercury, opiates, cannabis, barbiturates, amphetamines, phencyclidine.

### Virological and serological investigations of rabies in Cases 1, 2 and 3 (Fig 2)

**Fig 2:**
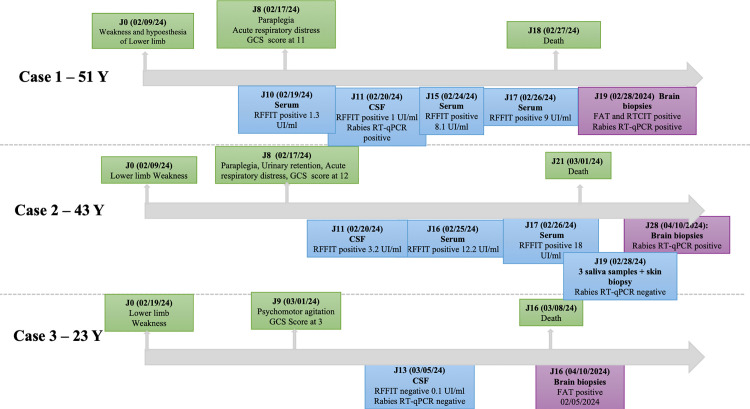
Timeline of the clinical evolution of the three cases. **The date of collection of the different samples.**.

Frozen skin biopsy and saliva samples were received only for Case 2(Fig 2). These samples collected at day 19 (D19) of the clinical evolution and one day before death were negative by RT-PCR.

The serum and CSF samples collected from Cases 1 and 2 showed the presence of rabies-specific antibodies and were indicative of rabies virus infection in unvaccinated people. The CSF sample obtained from Case 1 also tested positive by RT-PCR. The CSF sample collected from Case 3 was negative by RFFIT and RT-PCR.

**Fig 3:**
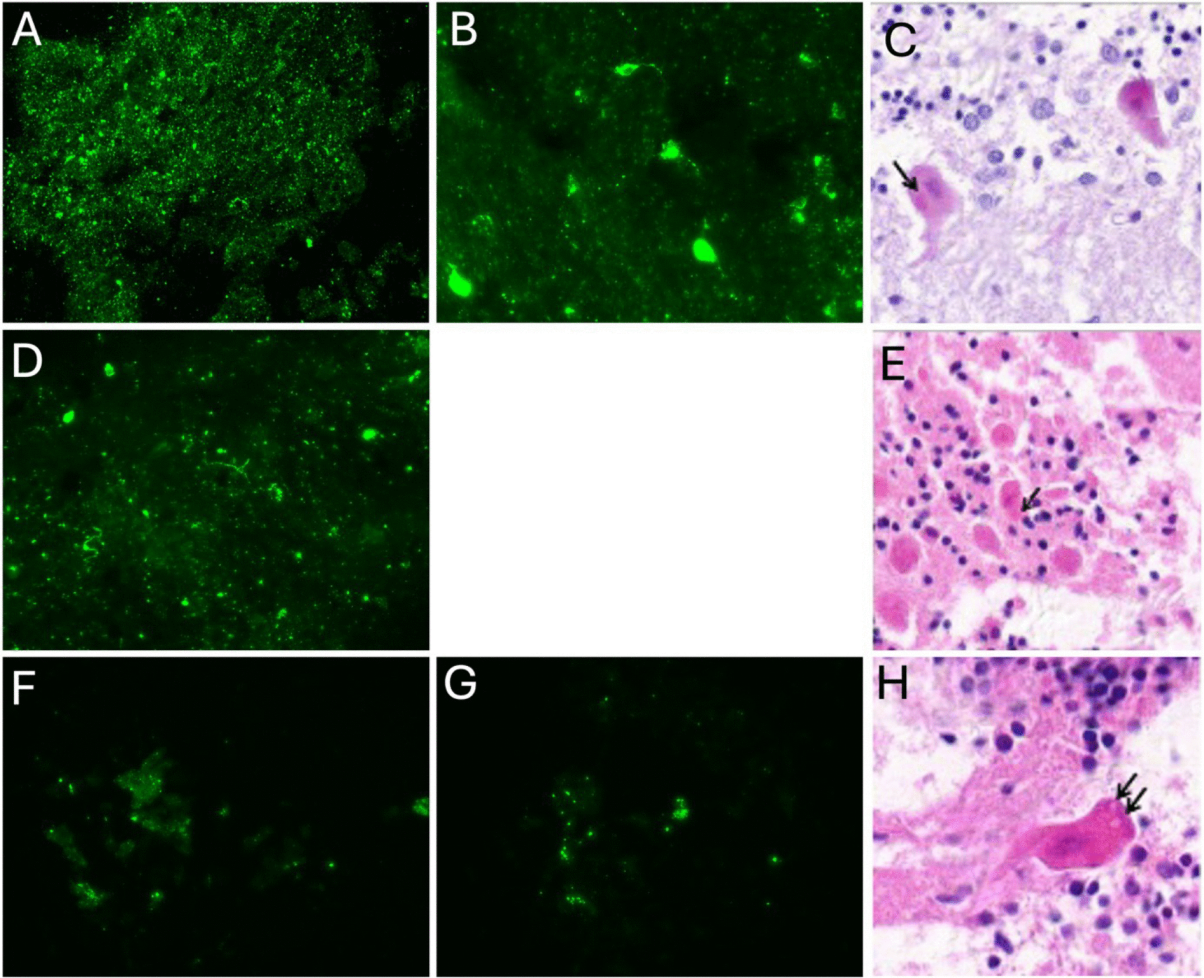
Fluorescent antibody test and analysis of histologicsection of different brain areas collected from the three cases. Fluorescent antibody test performed on brain areas of the three cases (A, B, D, F, G) and histological section of stained by Hematoxylin and Eosin “H&E” stain (C, E, H). Patient 1: A, cerebellum, magnification x40; B, cortex, magnification x40; C,cerebellum, magnification x150. Patient 2: D, hippocampus, magnification x40; cerebellum, resolution x100. Patient 3: F, hippocampus, magnification x40; G, brain stem, magnification x40; H, cerebellum, resolution x200. Black arrows show Negri bodies observed in rabies’s cases (intracytoplasmic eosinophilic, sharply outlined) pathognomonic inclusions in certain Purkinje neurons.

Analysis of brain biopsies of the occipital, parietal, frontal and temporal, cerebellum, hippocampus and brainstem confirmed a rabies virus infection in all cases (Fig 3).

### Characterization of the rabies virus strains infecting Cases 1, 2 and 3

The complete genome of the rabies virus was determined for Case 1 (independently from the cerebellum (sequence P1631) and from a non-specified area of the brain (Sequence P1632)) and Case 2 (from hippocampus, sequence P1642) (GenBank accession numbers PV606731, PV606730, PV606732, respectively). Their length and their genetic organization are consistent with that of previously sequenced vampire bat rabies virus genomes, with ﬁve open reading frames unidirectionally transcribed and separated by intergenic regions [34–37] and with the sequence previously obtained on Case 1 [37].

**Fig 4:**
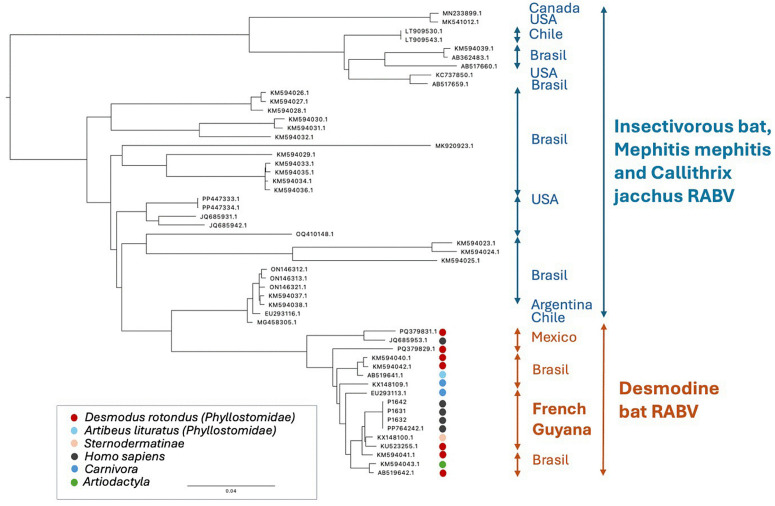
Phylogenetic analysis of rabies virus. Maximum likelihood phylogeny of rabies virus (RABV) from the Americas, highlighting host and geographic associations. The three sequences obtained from this study are located in the French Guyana cluster. The complete genome of the rabies virus was determined for Case 1 (independently from the cerebellum (sequence P1631) and from a non-specified area of the brain (Sequence P1632)) and Case 2 (from hippocampus, sequence P1642) (GenBank accession numbers PV606731, PV606730, PV606732, respectively). A color code indicates the species, order or family of the animal from which the virus was obtained.

The phylogenetic analysis of the sequences indicates that the rabies viruses responsible were similar to those circulating in hematophagous bats in this part of the world and closely related to those previously isolated from animals in French Guiana and the neighboring countries (Fig 4). The three sequences differ by a maximum of 3 nucleotides. For Case 3, only short sequences were obtained from the hippocampus and the brain stem (P1648; GenBank accession number PV978108). These partial sequences perfectly matched the two other whole genome sequences obtained from Cases 1 and 2. Therefore, bites by infected vampire bats seem by far the most likely route of transmission and our results are in favor of a unique virus circulating in the bat population living in the vicinity of the gold miners settlement at Eau Claire in January-February 2024, the most probable period of exposure of the three confirmed cases.

## Discussion

### Atypical presentation

Fifteen years after the first case of human rabies reported in French Guiana [38], we described three concurrent new cases of confirmed rabies presenting with a paralytic form, characterized by an atypical pattern of ascending bilateral motor deficit mimicking Guillain-Barré syndrome. The electrical analysis from the three cases was helpful to understand the pathophysiological mechanism of this infection (Tables 1, S1 and S2). Indeed, previous electrical and histological analysis of six rabies patients admitted to Thailand hospital (three furious forms and three paralytic forms) found progressive focal involvement at the bitten segment with dysfunction of the anterior horn cells in patients with a furious form and arguments for demyelinating neuropathy in paralytic patients, suggesting different neuropathogenic mechanisms [39]. Also, histological analysis of nerves from patients with the paralytic form confirms this hypothesis, showing segmental demyelination, remyelination, Wallerian degeneration, loss of myelinated fibers, and axonal loss [17]. However, electric studies of these three confirmed cases were different and showed a decrease in the amplitudes of motor potentials and normality of the amplitudes of sensory potentials consistent with pure motor axonal neuropathy as described in the furious forms [39]. The sensory deficit observed during the neurological examination in Case 1 and the symptoms of hypoesthesia in Cases 1 and 2 may suggest damage to the peripheral nerves or sensory ganglia. But this was not confirmed by electrical testing, possibly due to the early stage at which the ENMG was performed too early to detect axonal degeneration of the sensory nerves. Although inflammatory involvement with demyelination is suggested in paralytic forms [17], no demyelination was electrically found, and the search for antiganglioside antibodies was negative in all three cases. Clinically, these three reported cases were extremely difficult to differentiate from the axonal or myelin form of acute GBS. Electrophysiological examination may raise suspicion once anterior horn cell dysfunction has been detected.

Furthermore, the cases had a faster progression as compared to the typical paralytic forms, with altered consciousness occurring after eight days instead of the reported 11 days [40]. This rapid progression is more commonly found in furious forms. However, the other cardinal signs of furious form (fluctuating consciousness, hydrophobia or aerophobia, inspiratory spasms, signs of autonomic dysfunction) were not present in the three cases [40].

Moreover, CSF analysis was atypical and contributed to diagnosis wandering due to the marked hypercellularity, exceeding 100/mm³, which contrasted with the literature [41]. It is therefore possible that CSF pleocytosis is more common in cases linked to vampire bats than in other cases. This atypical number of elevated cells may also rule out typical GBS.

Although, to date, no link has been shown between different strains of the virus and clinical presentation [19], all three cases were infected with vampire bat rabies virus suggesting an intermediate form, between the furious and the paralytic, in terms of virus propagation mechanism and duration of evolution. The role of corticosteroid therapy –Cases 1 and 2 received betamethasone for low back pain (Table 1) –in this atypical form is questionable. Although this hypothesis seems to have been ruled out for the time being, it had been suggested that immunity might play a role in the development of the disease, given the increase in survival in immunocompromised mice and the greater cellular response in patients with the furious form [42]. More, a study comparing dogs with furious rabies to those with paralytic rabies suggests that, in its early stages, furious rabies is characterized by moderate inflammation and severe viral neuroinvasiveness. Paralyzing rabies is characterized by delayed viral neuroinvasion and more intense inflammation (lesions visible on MRI, detection of cytokines in the brain) than furious rabies [43].These cases emphasize the importance of considering vampire bat rabies virus in the presence of an atypical pattern of ascending motor deficit, with electrical analysis suggesting a motor neuronopathy resembling a motor variant of GBS [44], in areas exposed to vampire bat rabies virus. A more rapid consideration of rabies will allow to quickly perform targeted tests and discuss prophylaxis in exposed individuals even in the absence of bite marks.

These cases highlight the challenge of differential diagnosis in a context of complex biological and toxicological workup in people living in remote areas as in the Amazonian forest. The burden of rabies, a neglected tropical disease, in this region is greatly underestimated.

### Rabies on the Guiana Shield

The presence of vampire bat populations in many regions in Latin America and their recent territorial expansion [45] serve as a warning for epidemiological surveillance and for the potential veterinary and public health consequences. In the Guiana Shield area, nine cases of paralytic forms were reported in Guyana among gold miners [46] in the 1950s, five cases in Suriname in the 1970s [7], and numerous other cases likely occurred but were not detected or reported. In this country, vampire bat rabies is mainly maintained in the pristine forest habitats that provide sufficient food resources to allow vampire bats to survive and thus facilitating rabies transmission among bats [45,47]. However, although probably greatly underestimated, vampire bat rabies virus can also be transmitted to animals on the forest edge and in disturbed and anthropized areas. Between 1984 and 2024, 16 rabies cases were recorded in cattle (n = 10), bats (n = 1), and domestic carnivores (n = 5) on the coastal shore of French Guiana. The first case of human rabies was reported in 2008 [38], due to the a *Lyssavirus rabies* variant also associated with vampire bats [35].

### At-Risk Individuals

The three cases were gold miners living in the Amazon rainforest where a high prevalence of infection is found in bats [45]. People working in these illegal gold mining camps are primarily from Brazil and live in extremely poor conditions. They can be exposed to a variety of infectious diseases (e.g., malaria, leishmaniasis, leptospirosis), suffer from chronic conditions (high blood pressure, anemia, musculoskeletal disorders), and encounter wild fauna [47,48]. A recent survey revealed that among 539 people living in this area, 57% had been bitten by a bat in the past year [49]. Use of mosquito nets that would also protect humans from bat bites is rare (27%) [50]. Their remoteness from the health centers, which can be up to five days away by boat or on foot, severely limits their access to prevention and medical care [49]. In French Guiana, despite the presence of deforestation and farming activities, their scale remains minimal compared to the vast Amazon forest covering 90% of the territory. This allows the preservation of bat habitat and food availability, sustaining rabies virus circulation in vampire bat populations [45]. The concomitant symptomatology of these three cases suggests possible destruction of the bat’s habitat and possible attacks as described in northern Brazil, bordering French Guiana in 2004/2005 [8,51].

Thus, the hypothesis of rabies should be considered in patients who have stayed in Amazon rainforest, or returning from travel to high-risk areas, even in the absence of a history of bites and the negativity of laboratory tests performed in a first instance, as recorded in Case 3.

### Diagnosis difficulties

These cases highlight the major challenges of rabies laboratory diagnosis in remote Amazonian regions where people live in poor and isolated conditions, often far from health facilities. This issue is particularly acute among indigenous communities and workers in extractive industries –such as illegal miners and loggers, who live in precarious shelters and are frequently exposed to bats. In these outbreak, three laboratory-confirmed cases were identified. Laboratory analyses underscored the value of serological testing on CSF and serum, especially in atypical or prolonged clinical presentations differing from the classical “furious” form. *Post-mortem* brain biopsy remains the gold standard for definitive diagnosis in both humans and animals. Phylogenetic analysis of viral sequences obtained from these cases supported the hypotheses of vampire bat transmission as reported previously [38,45].

These findings raise serious issues about rabies prevention in such vulnerable populations. Human incidence may rise in the coming years due to deforestation and other anthropogenic activities that disrupt bat habitats and increase human exposure [11,45]. Bat bites are reportedly frequent, yet prevention and post-exposure prophylaxis (PEP) are mostly reactive public health measure, often implemented only after grouped cases or fatalities occur [12]. Although, human rabies is fully preventable through timely PEP administration following WHO recommendations [21], geographical isolation and logistical barriers frequently delay treatment, compromising its effectiveness [52]. In French Guiana, PEP is provided primarily to occupational at-risk populations such as animal handlers and wildlife professionals. The main anti-rabies treatment center, located in Cayenne (Fig 1), supplies vaccines and immunoglobulins following WHO protocols. However, its centralized location makes access extremely difficult for people from remote areas who must travel by plane, helicopter, canoe, or long overland routes – often without legal status, further complicating care. The three affected patients were illegal miners who had not accessed the healthcare system before entering the forest. To improve accessibility, vaccine support units (excluding immunoglobulins) have been established in regional and local hospitals (Kourou Hospital, Saint-Laurent du Maroni Hospital, Maripasoula, Grand Santi and Saint-Georges), implementing a shortened intradermal vaccination schedule to simplify PEP delivery in isolated communities. Pre-exposure vaccination should be encouraged in at-risk groups. Once exposed, it may no longer be possible to boost immunity. However, if the actual exposure that could occur in the future is insignificant, it could trigger an anamnestic response. Indeed, a study in Peru showed the presence of rabies antibodies in goats and sheep, suggesting widespread abortive infections in livestock in areas where vampire bat rabies is endemic [53].

## Conclusion

Vampire bat rabies virus is endemic in South America, particularly in the Amazon region, and is a constant threat to human populations. French Guiana, still preserved from deforestation, is conducive to maintaining rabies virus circulation in vampire bats. Disruption of this environment could increase human exposure to bat-transmitted rabies. The three cases described in our study illustrate the risk of under-reporting of human rabies and highlights the need for rabies laboratory confirmation to obtain accurate data on disease burden. The three confirmed cases had an atypical presentation, with a clinical picture suggestive of Guillain-Barré syndrome, making differential diagnosis difficult. Given the emergence of rabies virus and the peculiarities of the Amazon population, there is an urgent need to promote community awareness and implementation of prevention and One Health approaches to avoid this fatal issue.

## Supporting information

S1 Table Motor nerve conduction study of the 3 confirmed cases of rabies. The study of motor conduction at J12 for P1 at J12 for P2 J9 for P3. a-f: ankle to fibula head; CB: conduction block; DA: distal amplitude; DL: distal latency; f-k: fibular head to knee; MNV: Motor Nerve Velocity; m/s: meter per second; ms: millisecond; mV: millivolt; m/s: meter by second (··): no data.
(DOCX)

S2 Table Sensory nerve conduction study of the 3 confirmed cases of rabies. The study of sensitive conduction at J12 for P1 at J12 for P2 J9 for P3. µV: millivolt; m/s: meter by second.
(DOCX)

S1 Fig Cerebral and spinal MRI of Patient 3 at the comatose stage. First column: Diffusion-weighted sequence showing hypersignal in the frontal cortical ribbons on top and in internal capsule on bottom. Second column: Apparent Diffusion Coefficient (ADC) showing restricted diffusion in the frontal cortical ribbons on top and in internal capsule on bottom. Third column: FLAIR sequence showing hypersignal in the cortical ribbons on top and periaqueductal region on bottom. Fourth column: Spinal MRI T2 sequence showing pan-medullary transverse hypersignal. Arrows indicate hypersignal.
(TIF)
