# Supplementary material for: Paralytic rabies outbreak mimicking guillain–Barré syndrome in French Amazonia
Source: PLoS Negl Trop Dis. 2026 Mar 30;20(3):e0014149. doi: 10.1371/journal.pntd.0014149 (PMC13046258; doi:10.1371/journal.pntd.0014149)
Supplement: S1 Table — The study of motor conduction at J12 for P1 at J12 for P2 J9 for P3. a-f: ankle to fibula head; CB: conduction block; DA: distal amplitude; DL: distal latency; f-k: fibular head to knee; MNV: Motor Nerve Velocity; m/s: meter per second; ms: millisecond; mV: millivolt; m/s: meter by second (··): no data. (DOCX) [file pntd.0014149.s004.docx]

**S1 Table. Motor nerve conduction study of the 3 confirmed cases of rabies.**

|  | **Upper limbs** | | | | | | | | | | | | | | | | | | | | | | |
| --- | --- | --- | --- | --- | --- | --- | --- | --- | --- | --- | --- | --- | --- | --- | --- | --- | --- | --- | --- | --- | --- | --- | --- |
|  | **Right** | | | | | | | | | | |  | **Left** | | | | | | | | | | |
|  | **Median nerve** | | | | |  | **Ulnar nerve** | | | | |  | **Median nerve** | | | | |  | **Ulnar nerve** | | | | |
|  | **DL (ms)** | **DA (mV)** | **MNV (m/s)**  **w-e** | **CB** | **F wave (ms)** |  | **DL (ms)** | **DA (mV)** | **MNV (m/s)**  **w-e** | **CB** | **F wave (ms)** |  | **DL (ms)** | **DA (mV)** | **MNV (m/s)**  **w-e** | **CB** | **F wave (ms)** |  | **DL (ms)** | **DA (mV)** | **MNV (m/s)**  **w-e** | **CB** | **F wave (ms)** |
| ***Normal value*** | *4* | *4* | *46* | *··* | *32* |  | *3·5* | *4·8* | *46* | *··* | *32* |  | *4* | *4* | *46* | *··* | *32* |  | *3·5* | *4·8* | *46* | *··* | *32* |
| **P1** | 3·65 | 0·42 | 49 | ·· | ·· |  | 2·05 | 3·08 | 60.7 | no | ·· |  | 2·75 | 0·95 | 59 | ·· | ·· |  | 2·05 | 3·38 | 85·7 | no | ·· |
| **P2** | 3·65 | 0·01 | 77·8 | ·· | ·· |  | 2·28 | 1·43 | 68 | no | ·· |  | 2·4 | 0·5 | 65·7 | ·· | ·· |  | 2·05 | 1·03 | 51·2 | no | ·· |
| **P3** | 3·0 | 6·31 | 67·5 | no | ·· |  | 2·7 | 4·01 | 76·5 | no | ·· |  | 3·2 | 4·51 | 59·2 | ·· | ·· |  | 2·05 | 4·7 | 67·6 | no | ·· |

|  | **Lower limbs** | | | | | | | | | | | | | | | | | | | | | | | |
| --- | --- | --- | --- | --- | --- | --- | --- | --- | --- | --- | --- | --- | --- | --- | --- | --- | --- | --- | --- | --- | --- | --- | --- | --- |
|  | **Right** | | | | | | | | | | |  | **Left** | | | | | | | | | | | |
|  | **peroneal nerve** | | | | |  | **Tibial nerve** | | | | |  | **Peroneal nerve** | | | | |  | **Tibial nerve** | | | | | |
|  | **DL (ms)** | **DA (mV)** | **MNV (m/s)**  **a-f/f-k** | **CB** | **F wave (ms)** |  | **DL (ms)** | **DA (mV)** | **MNV (m/s)**  **a-f/f-k** | **CB** | **F wave (ms)** |  | **DL (ms)** | **DA (mV)** | **MNV (m/s)**  **a-f/f-k** | **CB** | **F wave (ms)** |  | **DL (ms)** | **DA (mV)** | **MNV (m/s)**  **a-f/f-k** | **CB** | **F wave (ms)** |  |
| ***Normal value*** | *5·6* | *2* | *40* | *··* | *55* |  | *6·5* | *5* | *40* | *··* | *55* |  | *5·6* | *2* | *40* | *··* | *55* |  | *6·5* | *5* | *40* | *··* | *55* |  |
| **P1** | 2·9 | 1·19 | 49·5 | no | ·· |  | 4·34 | 2·88 | ·· | ·· | ·· |  | 3·49 | 0·75 | ·· | ·· | ·· |  | 4·05 | 2·14 | ·· | ·· | ·· |  |
| **P2** | ·· | 0 | ·· | ·· | ·· |  | 5·82 | 0·1 | ·· | ·· | ·· |  | ·· | 0 | ·· | ·· | ·· |  | 3·02 | 0·46 | ·· | ·· | ·· |  |
| **P3** | ·· | 0 | ·· | ·· | ·· |  | 4·1 | 3·94 | ·· | ·· | ·· |  | 3·5 | 1·32 | 52·9 | ·· | ·· |  | 2·38 | 1·09 | 60·7 | ·· | ·· |  |

The study of motor conduction at J12 for P1 at J12 for P2 J9 for P3.

a-f: ankle to fibula head; CB: conduction block; DA: distal amplitude; DL: distal latency; f-k: fibular head to knee; MNV: Motor Nerve Velocity; m/s: meter per second; ms: millisecond; mV: millivolt; m/s: meter by second (··): no data
