# Supplementary material for: Paralytic rabies outbreak mimicking guillain–Barré syndrome in French Amazonia
Source: PLoS Negl Trop Dis. 2026 Mar 30;20(3):e0014149. doi: 10.1371/journal.pntd.0014149 (PMC13046258; doi:10.1371/journal.pntd.0014149)
Supplement: S2 Table — The study of sensitive conduction at J12 for P1 at J12 for P2 J9 for P3. µV: millivolt; m/s: meter by second. (DOCX) [file pntd.0014149.s001.docx]

**S2 Table. Sensory nerve conduction study of the 3 confirmed cases of rabies.**

|  | **Upper limbs** | | | | | | | | | | | |  | | **Lower limbs** | | | | | | |
| --- | --- | --- | --- | --- | --- | --- | --- | --- | --- | --- | --- | --- | --- | --- | --- | --- | --- | --- | --- | --- | --- |
|  | **Right** | | | | |  | **Left** | | | | | |  | | **Right** | | | | **Left** | | |
|  | **Median nerve** | |  | **Ulnar nerve** | |  | **Median nerve** | |  | **Ulnar nerve** | |  | | **Sural nerve** | | |  | **Sural nerve** | | |  |
|  | **DA (μV)** | **SNV (m/s)** |  | **DA (μV)** | **SNV (m/s)** |  | **DA (μV)** | **SNV (m/s)** |  | **DA (μV)** | **SNV (m/s)** |  | | **DA (μV)** | | **SNV (m/s)** |  | **DA (μV)** | | **SNV (m/s)** |  |
| ***Normal value*** | *7* | *47* |  | *5* | *48* |  | *7* | *47* |  | *5* | *48* |  | | *7* | | *41* |  | *7* | | *41* |  |
| **P1** | ·· | ·· |  | ·· | ·· |  | ·· | ·· |  | ·· | ·· |  | | ·· | | ·· |  | ·· | | ·· |  |
| **P2** | ·· | ·· |  | ·· | ·· |  | 12·1 | 73·5 |  | 4·9 | 70·9 |  | | 5·8 | | 57 |  | 5 | | 66·7 |  |
| **P3** | 11·4 | 75·8 |  | 11·4 | 68·2 |  | ·· | ·· |  | 6·2 | 62·1 |  | | 11·7 | | 55·8 |  | 20·2 | | 60·7 |  |

The study of sensitive conduction at J12 for P1 at J12 for P2 J9 for P3.

μV: millivolt; m/s: meter by second
